# Prevalence of Varicose Veins Among Surgeons: A Cross-Sectional Study

**DOI:** 10.7759/cureus.67687

**Published:** 2024-08-24

**Authors:** Turki A Qari, Khaled N Almatrafi, Fawaz R Khateb, Bader Al-Kaabi, Ahmed Al-Harbi, Samer Alabdali, Rashed Al-nemari, Sahar A Bannani

**Affiliations:** 1 Medicine and Surgery, Umm Al-Qura University, Makkah, SAU; 2 Surgery, Umm Al-Qura University, Makkah, SAU; 3 Surgical Oncology, King Abdullah Medical City, Makkah, SAU

**Keywords:** prevalence, vascular surgery, general surgery, surgeons, varicose veins

## Abstract

Background

Extended periods of standing, obesity, female sex, and older age are risk factors associated with a higher probability of developing varicose veins (VV). This study aimed to determine the prevalence of VV among surgeons in public hospitals in Makkah, Saudi Arabia, and to identify the risk factors associated with this condition.

Methodology

This is a descriptive cross-sectional study based on a validated online questionnaire distributed via hospital WhatsApp (Meta Platforms Inc., Menlo Park, CA) groups conducted between January and June 2024. The inclusion criteria of this study were surgeons of both sexes and any age group working in the selected hospitals in Makkah City, who agreed to participate in the study. The exclusion criteria were any other healthcare workers from outside the surgical field. We included 192 participants, and Epi Info software (Centers for Disease Control and Prevention, Atlanta, GA) was used to calculate the sample size. The data were collected, reviewed, and then fed into IBM SPSS Statistics software for Windows version 21 (IBM Corp., Armonk, NY). Every participant filled out a consent form. The university ethics committee of Umm Al-Qura University, Makkah, granted ethical permission.

Result

This study comprised 192 surgeons in Makkah hospitals. Twenty-eight surgeons were either diagnosed with VV or had signs of VV. Varicose veins were detected among 50% of thoracic surgeons, 42.9% of pediatric surgeons, and 26.3% of orthopedic surgeons. The most reported risk factors were a family history of VV (17%), hypertension (16%), and diabetes mellitus (14%). Surgeons aged 40 years or older had a higher prevalence of VV than younger ones. Also, 55.6% of obese surgeons had VV compared to 7.8% of others with average weight.

Conclusion

We found that VV is a common problem, particularly among individuals with predisposing factors, such as long standing hours, smoking, pregnancy, and obesity. We found that surgeons specializing in thoracic and pediatric specialties and female surgeons were more likely to be affected by VV. Preventive measures, such as avoiding prolonged standing, wearing compression stockings, and maintaining a healthy lifestyle, are recommended.

## Introduction

Worldwide, the prevalence of varicose veins (VV) ranges from 10% to 60% [1]. In Saudi Arabia, research has indicated that the occurrence of this condition is relatively high, estimated to be as much as 62%.

Furthermore, it has been observed that there is a yearly increase of around 5% in females and 2% in males [2, 3]. Valves can weaken and fail due to various factors, including venous thrombosis, heredity, and the deterioration of the venous wall's elastic tissue by chemical, mechanical, and enzymatic injuries interrupting blood vessel homeostasis [4,5]. The symptoms of the lower limb VV might be localized, affecting just that area, or they can be widespread, affecting the entire leg. While localized sensations like pain, burning, and itching are more common, other symptoms include leg pain, weariness, edema, and leg fatigue. Long durations of standing typically exacerbate symptoms; sitting and raising the legs of the patient alleviates these symptoms [6]. Complications from VV are not prevalent. However, clots in the deep veins of the legs may sometimes get bigger. These illnesses result in swelling and pain in the legs, which may require treatment when these symptoms are persistent. This increases morbidity and medical costs associated with these symptoms [7]. Because surgeons must stand and sit for extended periods during surgical operations, they are more likely to acquire VV. The main objectives of this study were to determine how common varicose veins are among surgeons in Makkah City, Saudi Arabia, and what risk factors are linked to this condition.

## Materials and methods

Study design and criteria

A descriptive cross-sectional study based on a validated, self-administered online questionnaire conducted between January 2024 and June 2024 targeted surgeons of both male and female genders from different specialties in the public hospitals of Makkah. The inclusion criteria for the participating individuals were surgeons of both sexes, any age group, any nationality, with different years of employment, working in the selected hospitals, and those who had agreed to participate in the study. The exclusion criteria were any other healthcare workers from outside the surgical field, outside the selected hospitals in Makkah, or those who refused to participate.

Procedure and assessments

The items in the questionnaire were taken from earlier literature done in Saudi Arabia [8]. A pilot study with 25 individuals was conducted. To evaluate validity and reliability, the pilot study's findings were reviewed. The questionnaire covered participants' socio-demographic characteristics, such as gender, age, nationality, specialty, and years of working in the field. The second section covered lifestyle and work-related variables. The last section included participants' health-related data (comorbidities, hormonal therapy/contraceptives, etc.). Before starting the questionnaire, consent was obtained from all participants, and we excluded those who did not agree to participate in our survey. Data were gathered using Google Forms (Google Inc., Mountain View, CA) and an online, verified survey. Several potential biases could affect the study's outcomes. Selection bias may have occurred due to the convenience sampling method used, as the survey was distributed only via hospital WhatsApp (Meta Platforms Inc., Menlo Park, CA) groups, potentially excluding surgeons who were not part of these groups or were less active on WhatsApp. Response bias might be present if the surgeons who chose to participate differed significantly from those who did not, such as being more interested in the topic or having more free time to complete the survey. Information bias could arise from self-reported data, as participants might inaccurately report their socio-demographic or health-related information. However, it is important to note that we made significant efforts to mitigate these biases. We ensured anonymity and confidentiality and encouraged honest responses, all of which contributed to the credibility of our study. The participants were told about the aim of the study and the voluntary nature of their participation. The study questionnaire was available to participants until no more new answers were needed.

Ethical consideration and sample size calculation

Epi Info software (Centers for Disease Control and Prevention, Atlanta, GA) was used to calculate the sample size. The sample size calculator showed that 190 was the least suggested size for this study, with a 50% response distribution, a 5% margin of error, and a 95% confidence interval level. In addition to obtaining institutional review board (IRB) approval, several ethical considerations were addressed, underscoring our commitment to the integrity and ethical conduct of the study. Informed consent was obtained from all participants before they started the questionnaire. The consent process included informing participants about the study's purpose, the voluntary nature of their participation, and their right to withdraw at any time without any repercussions. Data confidentiality was strictly maintained; all responses were anonymized, and no personally identifiable information was collected. The data were stored securely, accessible only to the research team, and used solely for this study. Institutional Review Board approval was obtained from Umm Al-Qura University's Biomedical Research Ethics Committee with approval no. HAPO-02-K-012-2023-09-1733.

Statistical analysis

The data were collected, reviewed, and then fed into IBM SPSS Statistics software for Windows version 21 (IBM Corp., Armonk, NY). All statistical methods used were two-tailed with an alpha level of 0.05, considering significance if the p-value was less than or equal to 0.05. Descriptive analysis was done by prescribing frequency distribution and percentage for study variables, including the surgeons' personal and work data. Surgeons' habits, risk factors of VV, reported practices to prevent VV, and overall prevalence of VV were graphed. Cross-tabulation to assess the relationship between having VV and surgeons' personal data and work data, besides relationships with other factors, was carried out with Pearson's chi-square test for significance and Fisher's exact probability test if there were small frequency distributions.

## Results

A total of 192 eligible surgeons in Makkah hospitals were included. Surgeons' ages ranged from 24 to 55 years, with a mean age of 33.8 ± 7.0 years old. One hundred and thirty-six (70.8%) surgeons were male, and 171 (89.1%) were Saudi nationals. As for the body mass index, 116 (60.4%) had average body weight, 67 (34.9%) were overweight, and nine (4.7%) were obese. A total of 100 (52.1%) surgeons were married, and 84 (43.8%) were single (Table 1).

**Table 1:** Personal characteristics of sampled surgeons from the public hospitals of Makkah, Saudi Arabia

Personal data	Frequency (%)
Age, in years	
< 30	67 (34.9%)
30-39	83 (43.2%)
40+	42 (21.9%)
Mean ± SD	33.8 ± 7.0
Gender	
Male	136 (70.8%)
Female	56 (29.2%)
Body mass index	
Normal weight	116 (60.4%)
Overweight	67 (34.9%)
Obese	9 (4.7%)
Nationality	
Saudi	171 (89.1%)
Non-Saudi	21 (10.9%)
Marital status	
Single	84 (43.8%)
Married	100 (52.1%)
Divorced	8 (4.2%)
Number of children	
1-2	37 (40.7%)
3-4	41 (45.1%)
5+	13 (14.3%)
Mean ± SD	2.1 ± 1.1

Regarding specialties, 38.5% were general surgeons, 16.7% were obstetricians and gynecologists, 9.9% were orthopedic surgeons, 7.3% were pediatric surgeons, and 6.8% were neurosurgeons. The least reported specialties were vascular surgery (2.1%), colorectal surgery (1.6%), and ophthalmology (1.6%). As for the number of work years, 41.7% had worked for one to four years, and 26% worked for 10-19 years. A total of 17.2% were used to standing in surgery for less than two hours, 43.8% stood in surgery for two to four hours, and 10.4% stood for more than eight hours. On the other hand, most of the surgeons (85.9%) sat in surgery for less than two hours, and 12% sat for two to four hours. Only 22.4% reported they usually lift objects that were 23 kg or more during work time, 33.3% were smokers, and 52.1% frequently practiced sports or physical exercise less than four times a week (Table 2).

**Table 2:** Surgeons' work data and habits

Work data	Frequency (%)
Specialty	
General surgeon	74 (38.5%)
Obstetrician and gynecologist	32 (16.7%)
Orthopedic surgeon	19 (9.9%)
Pediatric surgeon	14 (7.3%)
Neurosurgeon	13 (6.8%)
Otolaryngologist (ENT)	11 (5.7%)
Urologist	11 (5.7%)
Thoracic surgeon	8 (4.2%)
Vascular surgeon	4 (2.1%)
Colon and rectal surgeon	3 (1.6%)
Ophthalmologist	3 (1.6%)
Years working in the field	
1-4	80 (41.7%)
5-9	44 (22.9%)
10-19	50 (26.0%)
20+	18 (9.4%)
How many hours do you stand in a surgery?	
< 2 hours	33 (17.2%)
2-4 hours	84 (43.8%)
4-6 hours	37 (19.3%)
6-8 hours	18 (9.4%)
> 8 hours	20 (10.4%)
How many hours do you sit in a surgery?	
< 2 hours	165 (85.9%)
2-4 hours	23 (12.0%)
4-6 hours	2 (1.0%)
Do you usually lift objects that are 23 kg or more during work time?	
Yes	43 (22.4%)
No	149 (77.6%)
Are you a smoker?	
Yes	64 (33.3%)
No	128 (66.7%)
Do you frequently practice sports or physical exercise?	
None	75 (39.1%)
< 4 times per week	100 (52.1%)
> 7 times per week	17 (8.9%)

**Figure 1:**
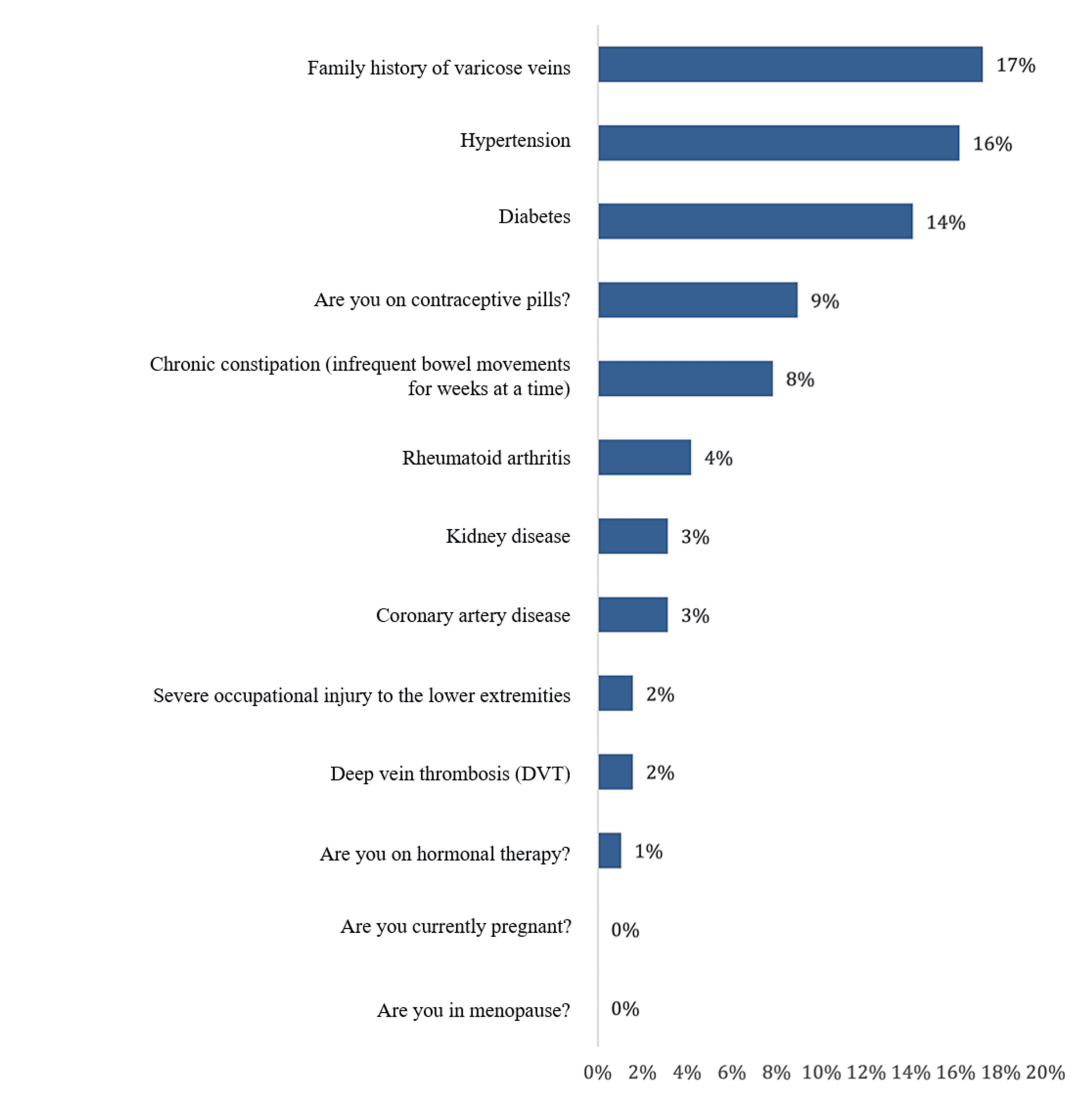
Risk factors and determinants of varicose veins among the surgeons in the study

Concerning the prevalence of VV among the surgeons in the study, a total of 28 (14.6%) were either diagnosed with VV or had signs of VV, while 164 (85.4%) had normal veins. The most reported risk factors were family history of VV (17%), hypertension (16%), diabetes mellitus (14%), being on contraceptive pills (for females; 9%), chronic constipation (8%), and rheumatoid arthritis (4%) (Figure 1).

**Figure 2:**
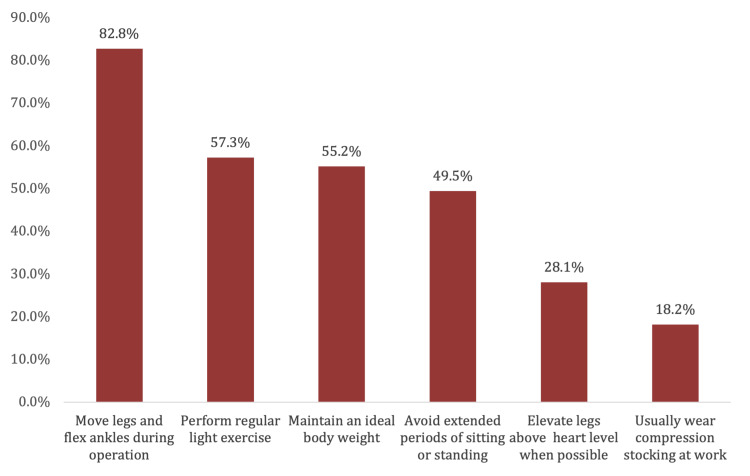
Practices reported by surgeons to prevent varicose veins

The most reported preventive practices were moving the legs and flexing the ankles during operations (82.8%), performing regular light exercise (57.3%), maintaining an ideal body weight (55.2%), avoiding extended periods of sitting or standing (49.5%), elevating legs above heart level when possible (28.1%), and wearing compression stockings at work (18.2%) (Figure 2).

Approximately 28.6% of surgeons aged 40 years or older had VV versus 10.4% of younger surgeons (P = 0.015). Also, 55.6% of obese surgeons had VV compared to 7.8% of those with average weight (P = 0.001) (Table 3).

**Table 3:** The correlation between having varicose veins and surgeons' personal data

Personal data	Having varicose veins		p-value
	Yes	No	
	Frequency (%)	Frequency (%)	
Age, in years			.015*
< 30	7 (10.4%)	60 (89.6%)	
30-39	9 (10.8%)	74 (89.2%)	
40+	12 (28.6%)	30 (71.4%)	
Gender			.085
Male	16 (11.8%)	120 (88.2%)	
Female	12 (21.4%)	44 (78.6%)	
Body mass index			.001*
Normal weight	9 (7.8%)	107 (92.2%)	
Overweight	14 (20.9%)	53 (79.1%)	
Obese	5 (55.6%)	4 (44.4%)	
Nationality			.010*^
Saudi	21 (12.3%)	150 (87.7%)	
Non-Saudi	7 (33.3%)	14 (66.7%)	
Marital status			.168
Single	8 (9.5%)	76 (90.5%)	
Married	18 (18.0%)	82 (82.0%)	
Divorced	2 (25.0%)	6 (75.0%)	
Number of children			.947
1-2	7 (18.9%)	30 (81.1%)	
3-4	8 (19.5%)	33 (80.5%)	
5+	3 (23.1%)	10 (76.9%)	

Varicose veins were significantly associated among 50% of thoracic surgeons, 42.9% of pediatric surgeons, 26.3% of orthopedic surgeons, and 18.2% of otolaryngologists (ENT) (P = 0.002). Also, 44.4% of surgeons with 20 years or more of experience had significantly associated VV compared to 13.8% of those with one to four years of experience (P = 0.002). Varicose veins were significantly associated among 23.3% of those who usually lifted objects that were 23 kg or more during work time versus 12.1% of those who did not (P = 0.049) (Table 4).

**Table 4:** The correlation between surgeons' work data and their personal data

Work data	Having varicose veins			p-value
	Yes	No		
	Frequency (%)	Frequency (%)		
Specialty				.002*^
Colon and rectal surgeon	0 (0.0%)		3 (100.0%)	
General surgeon	5 (6.8%)		69 (93.2%)	
Neurosurgeon	0 (0.0%)		13 (100.0%)	
Obstetrician and gynecologist	5 (15.6%)		27 (84.4%)	
Ophthalmologist	0 (0.0%)		3 (100.0%)	
Orthopedic surgeon	5 (26.3%)		14 (73.7%)	
Otolaryngologist [ENT]	2 (18.2%)		9 (81.8%)	
Pediatric surgeon	6 (42.9%)		8 (57.1%)	
Thoracic surgeon	4 (50.0%)		4 (50.0%)	
Urologist	1 (9.1%)		10 (90.9%)	
Vascular surgeon	0 (0.0%)		4 (100.0%)	
Years working in the field				.002*
1-4 years	11 (13.8%)		69 (86.3%)	
5-9 years	4 (9.1%)		40 (90.9%)	
10-19 years	5 (10.0%)		45 (90.0%)	
20+ years	8 (44.4%)		10 (55.6%)	
How many hours do you stand in a surgery?				.268^
< 2 hours	5 (15.2%)		28 (84.8%)	
2-4 hours	8 (9.5%)		76 (90.5%)	
4-6 hours	9 (24.3%)		28 (75.7%)	
6-8 hours	2 (11.1%)		16 (88.9%)	
> 8 hours	4 (20.0%)		16 (80.0%)	
How many hours do you sit in a surgery?				.099^
< 2 hours	20 (12.1%)		145 (87.9%)	
2-4 hours	7 (30.4%)		16 (69.6%)	
4-6 hours	1 (50.0%)		1 (50.0%)	
6-8 hours	0 (0.0%)		1 (100.0%)	
> 8 hours	0 (0.0%)		1 (100.0%)	
Do you usually lift objects that are 23 kg or more during work time?				.049*
Yes	10 (23.3%)		33 (76.7%)	
No	18 (12.1%)		131 (87.9%)	
Are you a smoker?				0.112
Yes	13 (20.3%)		51 (79.7%)	
No	15 (11.7%)		113 (88.3%)	
Do you frequently practice sports or physical exercise?				0.09
No	16 (21.3%)		59 (78.7%)	
< 4 times per week	11 (11.0%)		89 (89.0%)	
> 7 times per week	1 (5.9%)		16 (94.1%)	

Among all included factors, VV was significantly associated higher among those with a family history of VV (36.4% vs. 10.1%, P = 0.001), deep vein thrombosis (66.7% vs. 13.8%, P = 0.010), hypertension (32.3% vs. 11.2%, P = 0.002), rheumatoid arthritis (50% vs. 13%, P = 0.004), and contraceptive use (60% vs. 17.6%, P = 0.028). The condition of VV was significantly associated with lower among surgeons performing regular light exercise (10% vs. 20.7%, P = 0.037) and those maintaining an ideal body weight (8.5% vs. 22.1%, P = 0.008) (Table 5).

**Table 5:** Other factors associated with the occurrence of varicose veins among surgeons

Other factors		Having varicose veins		p-value
		Yes	No	
		Frequency (%)	Frequency (%)	
Family history of varicose veins	Yes	12 (36.4%)	21 (63.6%)	.001*
	No	16 (10.1%)	143 (89.9%)	
Deep vein thrombosis (DVT)	Yes	2 (66.7%)	1 (33.3%)	.010*
	No	26 (13.8%)	163 (86.2%)	
Coronary artery disease	Yes	1 (16.7%)	5 (83.3%)	.883
	No	27 (14.5%)	159 (85.5%)	
Hypertension	Yes	10 (32.3%)	21 (67.7%)	.002*
	No	18 (11.2%)	143 (88.8%)	
Chronic constipation (infrequent bowel movement for weeks at a time)	Yes	6 (40.0%)	9 (60.0%)	.004*
	No	22 (12.4%)	155 (87.6%)	
Diabetes	Yes	7 (25.9%)	20 (74.1%)	.072
	No	21 (12.7%)	144 (87.3%)	
Kidney disease	Yes	2 (33.3%)	4 (66.7%)	.186
	No	26 (14.0%)	160 (86.0%)	
Rheumatoid arthritis	Yes	4 (50.0%)	4 (50.0%)	.004*
	No	24 (13.0%)	160 (87.0%)	
Severe occupational injury to the lower extremities	Yes	1 (33.3%)	2 (66.7%)	.354
	No	27 (14.3%)	162 (85.7%)	
Are you on hormonal therapy?	Yes	1 (50.0%)	1 (50.0%)	.154
	No	27 (14.2%)	163 (85.8%)	
Are you on contraceptive pills?	Yes	3 (14.2%)	2 (40.0%)	.028*
	No	9 (17.6%)	42 (82.4%)	
Do you usually wear compression stockings at work?	Yes	6 (17.1%)	29 (82.9%)	.635
	No	22 (14.0%)	135 (86.0%)	
Do you elevate your legs above your heart when possible?	Yes	7 (13.0%)	47 (87.0%)	.691
	No	21 (15.2%)	117 (84.8%)	
Do you move your legs and flex your ankles during the operation?	Yes	21 (13.2%)	138 (86.8%)	.236
	No	7 (21.2%)	26 (78.8%)	
Do you perform regular light exercise?	Yes	11 (10.0%)	99 (90.0%)	.037*
	No	17 (20.7%)	65 (79.3%)	
Do you avoid extended periods of sitting or standing?	Yes	16 (16.8%)	79 (83.2%)	.380
	No	12 (12.4%)	85 (87.6%)	
Do you maintain an ideal body weight?	Yes	9 (8.5%)	97 (91.5%)	.008*
	No	19 (22.1%)	67 (77.9%)	

## Discussion

Varicose veins are a condition involving the lower limbs, a common problem affecting a portion of the general population [9], especially those who have predisposing factors for VV, such as obesity, smoking, pregnancy, and long hours spent standing [10-12].

In our study, 28 surgeons were either diagnosed with VV or had signs of VV. One known risk factor for venous disease development is older age; the prevalence of venous illness rises with aging. In line with this, 28.6% of surgeons aged 40 years or more had VV versus 10.4% of surgeons aged less than 40 years. These results are similar to a previous study conducted in Abha, Saudi Arabia, where the most common age group with VV was 36 to 45 years old [13]; this may be brought on by a weakening of the calf muscles, which over time causes the vessel walls to deteriorate gradually and increases pressure on superficial veins [14]. Our study found the highest prevalence of VV in thoracic surgeons (50%), followed by pediatric surgeons (42.9%), and orthopedic surgeons (26.3%); a potential association exists between prolonged periods of intraoperative standing and the observed outcomes. Conversely, the lowest VV prevalence was in vascular and colorectal surgeons (0%). This could be attributed to vascular surgeons' extensive knowledge and expertise regarding VV, leading to heightened awareness and proactive preventative measures.

In this study, there was a noteworthy distinction in the diagnosis of VV between male and female surgeons, with 11.8% of males and 21.4% of females diagnosed. Gender is one of the contributing factors for VV [15]. Likewise, several earlier studies found that women are more likely than men to develop VV [16-18]. The hormones progesterone and estrogen may impact women's propensity to develop VV. When progesterone attaches to its receptor on the venous walls, it decreases collagen synthesis and causes the smooth muscle in the veins to become hypotonic [19].

Most of the surgeons involved in our study would stand for two to four hours in a surgery (n = 84). The results showed that 9.5% of those surgeons were diagnosed with VV. For surgeons who stood for more than eight hours in a surgery (n = 20), 20% were diagnosed with VV, which is the highest percentage. In a study by Krijnen et al., standing position was an exacerbating factor for VV in the European population [20]. Nia et al. found that nurses who stand for longer than four hours had four times higher odds of developing VVs compared to those who stand for shorter periods [21].

Exercise strengthens the lower limb muscles, particularly the gastrocnemius, which has an excellent pumping action. Additionally, regular physical activity keeps blood flow healthy and prevents venous stasis [13]. Our study showed that individuals who regularly performed light exercise for at least four hours per week showed a lower propensity to develop VV than individuals who were either irregular or did not exercise. Additionally, surgeons reported some practices to prevent VVs. The most common preventive practices were moving their legs and flexing ankles during operation, avoiding prolonged sitting or standing, and wearing compression stockings while at work.

According to our study, the diagnosis rate for surgeons with a family history of VV was 36.4%, which is much higher than the rate for surgeons without a family history (10.1%). Although specific genes are yet unknown, several studies have shown that family history is a significant risk factor for vein disorders. [22-24].

Strengths

This study provides valuable insights into the prevalence of VV among surgeons in Makkah, Saudi Arabia. We consider it a large city and include a lot of surgeons and a wide range of specialties, so we identified those most affected and developed targeted prevention plans.

Limitations

One of the study's limitations is the small sample size. The research on changes in VV over time was not possible due to the cross-sectional design. Furthermore, we were unsuccessful in determining the individuals' workout and lifestyle routines. Moreover, because this study only included surgeons from one city, its conclusions might not apply to other demographics.

## Conclusions

This cross-sectional study found that VV in Makkah is a common problem, particularly among individuals with predisposing factors such as obesity, smoking, pregnancy, and standing for long hours. Additionally, age, gender, duration of standing during surgery, and family history were identified as significant factors associated with VV diagnoses. We found that surgeons specializing in thoracic and pediatric specialties and female surgeons were more likely to have VV. The study emphasizes the importance of increased awareness, education, and preventive measures to address VV among surgeons. We recommend that surgeons at risk for VV take precautions, such as reducing standing time in the operating room, bringing chairs in the operating room, wearing compression stockings, and engaging in leg movement when standing is necessary. Adopting a healthy lifestyle is also advised. Further research and initiatives are needed to enhance prevention and management strategies for this condition.
